# 288 Propranolol Use in Adult Burn Patients: Results from a Multi-center Safety and Efficacy Trial

**DOI:** 10.1093/jbcr/irad045.263

**Published:** 2023-08-29

**Authors:** David Herndon, Joshua Carson, Shawn P Fagan, Celeste Finnerty, Kevin N Foster, Richard Gamelli, Nicole S Gibran, Jeremy Goverman, David T Harrington, Doug Hayden, James H Holmes, Marc Jeschke, Matthew Klein, David Mozingo, Cathryn Oldmixon, Tam N Pham, David Shoenfeld, Charles E Wade, Lucy Wibbenmeyer, Steven E Wolf

**Affiliations:** CEO, Joseph Still Burn Research Foundation, Senior Editor Journal of Burn Care and Research, Augusta, Georgia; Loyola University Medical Center, Maywood, Illinois; JMS Burn Center, Augusta, Georgia; The University of Texas Medical Branch, Galveston, Texas; AZ Burn Center at Valleywise Health, Cave Creek, Arizona; Loyola University School of Medicine, Maywood, Illinois; University of Washington, Seattle, Washington; Massachusetts General Hospital, Boston, Massachusetts; Rhode Island Hospital/Brown Univeristy, Providence, Rhode Island; Massachusetts General Hospital, Boston, Massachusetts; WFSOM, Winston-Salem, North Carolina; Hamilton Health Sciences, Toronto, Ontario; PTC Therapeutics, South Plainfield, New Jersey; University of Florida College of Medicine, Gainesville, Florida; Massachusetts General Hospital, Boston, Massachusetts; University of Washington, Seattle, Washington; Massachusetts General Hospital, Boston, Massachusetts; Center for Translational Injury Research, University of Texas, Houston, Texas; University of Iowa, Iowa City, Iowa; The University of Texas Medical Branch, Galveston, Texas

## Abstract

**Introduction:**

The hypermetabolic response induced by a large burn is associated with increased cardiac work, total body wasting, and impaired physical function, leading to delays in psychosocial recovery and reintegration into society, and persistence of chronic conditions. Modulation of this response could reduce long-term burn morbidity. Catecholamines drive burn-induced hypermetabolism, therefore clinicians have employed the non-selective beta-adrenergic receptor blocker propranolol to reduce cardiac stress and the hypermetabolic response. Here, in a masked, placebo-controlled study, we investigated the safety and efficacy of the administration of propranolol to adult burn patients during the acute hospitalization period.

**Methods:**

Adults with large burns (n=157, mean 38 ± 15% TBSA) were enrolled at 13 participating centers. Subjects were randomized to receive placebo (n=75) or propranolol (n=82) with data collected for 72 and 79 subjects, respectively. Propranolol administration was initiated with an oral dose of 0.2 mg/kg dose and adjusted to decrease mean heart rate by 20%. The primary endpoint was effect on cardiac rate pressure product (CRPP), while the secondary endpoints included infections and survival. Adverse events were also tracked. Assessments were conducted between the time of consent and discharge, with a long-term follow-up visit occurring approximately 5 months post-burn on average.

**Results:**

CRPP was significantly reduced with propranolol during the acute hospitalization period (p< 0.0001, Fig 1). The initial dose of propranolol adequately reduced heart rate in most patients. Infections were not different between cohorts. Cardiac complications (atrial arrhythmias, ventricular arrhythmias, cardiac arrest) occurred at similar rates for each group, as did additional complications (abdominal compartment syndrome, deep vein thromboses, pneumothorax, pulmonary embolus). Mortality was similar for both the placebo and propranolol cohorts. Post-discharge heart rates and blood pressures were similar in both groups at approximately 5 months following injury.

**Conclusions:**

This study demonstrates that the short-term administration of propranolol to adults with large burns decreases CRPP without increasing complications, infections, or mortality. Furthermore, a safe dosing strategy was identified. This strategy may be effective for reducing acute cardiac stress and hypermetabolism. Future studies should focus on the effects of propranolol administration for longer periods, determining the effects on additional endpoints like the hypermetabolic response, pathologic scarring, long-term cardiac dysfunction, whether smaller burns benefit from the administration of propranolol.

